# Oral delivery of bi-autoantigens by bacterium-like particles (BLPs) against autoimmune diabetes in NOD mice

**DOI:** 10.1080/10717544.2023.2173339

**Published:** 2023-01-31

**Authors:** Ruifeng Mao, Jin Wang, Ying Xu, Yuqi Wang, Mengmeng Wu, Lixia Mao, Yingying Chen, Dengchao Li, Tong Zhang, Enjie Diao, Zhenjing Chi, Yefu Wang, Xin Chang

**Affiliations:** a Jiangsu Collaborative Innovation Center of Regional Modern Agriculture & Environmental Protection, School of Life Sciences, Huaiyin Normal University, Huai’an223300, China; b Nanjing Lishui People’s Hospital, Zhongda Hospital Lishui Branch, Southeast University, Nanjing211200, China; c Huai’an First People’s Hospital, Nanjing Medical University, Huai’an223300, China; d State Key Laboratory of Virology, College of Life Sciences, Wuhan University, Wuhan430072, China

**Keywords:** Type 1 diabetes mellitus, immune tolerance, autoantigen, bacterium-like particles, oral vaccination

## Abstract

Induction of oral tolerance by vaccination with type 1 diabetes mellitus (T1DM)-associated autoantigens exhibits great potential in preventing and treating this autoimmune disease. However, antigen degradation in the gastrointestinal tract (GIT) limits the delivery efficiency of oral antigens. Previously, bacterium-like particles (BLPs) have been used to deliver a single-chain insulin (SCI-59) analog (BLPs-SCI-59) or the intracellular domain of insulinoma-associated protein 2 (IA-2ic) (BLPs-IA-2ic). Both monovalent BLPs vaccines can suppress T1DM in NOD mice by stimulating the corresponding antigen-specific oral tolerance, respectively. Here, we constructed two bivalent BLPs vaccines which simultaneously deliver SCI-59 and IA-2ic (Bivalent vaccine-mix or Bivalent vaccine-SA), and evaluated whether there is an additive beneficial effect on tolerance induction and suppression of T1DM by treatment with BLPs-delivered bi-autoantigens. Compared to the monovalent BLPs vaccines, oral administration of the Bivalent vaccine-mix could significantly reduce morbidity and mortality in T1DM. Treatment with the bivalent BLPs vaccines (especially Bivalent vaccine-mix) endowed the mice with a stronger ability to regulate blood glucose and protect the integrity and function of pancreatic islets than the monovalent BLPs vaccines treatment. This additive effect of BLPs-delivered bi-autoantigens on T1DM prevention may be related to that SCI-59- and IA-2-specific Th2-like immune responses could be induced, which was more beneficial for the correction of Th1/Th2 imbalance. In addition, more CD4^+^CD25^+^Foxp3^+^ regulatory T cells (Tregs) were induced by treatment with the bivalent BLPs vaccines than did the monovalent BLPs vaccines. Therefore, multiple autoantigens delivered by BLPs maybe a promising strategy to prevent T1DM by efficiently inducing antigen-specific immune tolerance.

## Introduction

As a chronic autoimmune disease, type 1 diabetes mellitus (T1DM) mainly presents in children and adolescents. However, it may occur at any age. It has been reported that T1DM prevalence performs an increasing trend in recent years (Kanta et al., 2020; Patterson et al., 2014). In T1DM, insulin-producing pancreatic β cells are recognized and destroyed by the self-reactive immune responses, resulting in insulin deficiency, and therefore, T1DM patients require lifelong therapy with exogenous insulin (Katsarou et al., 2017). The etiology of T1DM is complicated and multifactorial, including genetic, immunological, epigenetic, environmental, physical, and social factors, resulting in the breakdown of autoimmune tolerance (Zajec et al., 2022; Chwalba et al., 2021; Bluestone et al., 2010; Bauer et al., 2021). However, the detailed underlying mechanisms have not been fully elucidated and remain to be further explored. As the primary pathological presentation of T1DM, insulitis results from the infiltration of multiple immune cells, including CD4 and CD8 T cells, B cells, as well as macrophages (Peakman, 2013). The initial laboratory presentation of the self-reactive immune response against pancreatic β cells is the occurrence of T1DM-related autoantibodies, which seem to be a key marker rather than a cause of T1DM-associated autoimmunity. Several islet autoantibodies have been identified, and can be employed in the prediction, diagnosis, or prognostic of T1DM (Lampasona & Liberati, 2016). Therefore, as a predictable disease (Simmons & Michels, 2015), there is an opportunity to enable the development of therapies, such as immunotherapy, to prevent and/or modify T1DM (Dayan et al., 2021; Bluestone et al., 2021). One important goal of immunotherapy is to induce or restore immune tolerance to T1DM-related autoantigens prior to and/or at disease onset.

Antigen-specific immunotherapy, which refers to vaccination with disease-associated antigens regardless of the mode of administration and the corresponding mechanism of action, exhibits a potential role in restoring self-tolerance in autoimmune diseases, including T1DM (Serra & Santamaria, 2019; Kreiner et al., 2021). Oral immune tolerance, which refers to the induction of peripheral tolerance by prior oral antigen administration, is featured by the specific suppression of immune responses to this antigen as a result of prior oral exposure (Rezende & Weiner, 2017). This process has extremely immunological importance because it contributes to maintaining host homeostasis between exogenous antigens and the self-components. Once it fails to develop oral tolerance, a cascade of adverse reactions, including food allergies, inflammatory bowel diseases, infections as well as autoimmune diseases, may be induced (Wambre & Jeong, 2018). The mechanism responsible for oral tolerance has been extensively explored, and the related decisive factors include the type, dose, consumption time, and administration form of the antigen(s) (Wambre & Jeong, 2018; Rezende & Weiner, 2022; Tordesillas & Berin, 2018; Sricharunrat et al., 2018). Regarding the dose of the administered antigen, high doses lead to deletion and/or anergy of reactive T cells specific for this antigen, whereas low and repeat doses promote the production of regulatory T cells (Tregs) (Rezende & Weiner, 2022; Tordesillas & Berin, 2018). As it can induce systemic and local unresponsiveness to the administered antigen(s), studies aiming to induce antigen-specific tolerance have been intensively performed to prevent and treat various diseases, particularly allergies and autoimmune diseases, including T1DM (Sricharunrat et al., 2018; Mao et al., 2020; Mao et al., 2020).

However, the digestion of the orally administered antigen in the stomach by acids and/or enzymes is challenging for oral tolerance induction. Oral vaccination with 5 mg native human insulin (twice a week for a total of 10 doses) had no significant effect in delaying the development of T1DM in non-obese diabetic (NOD) mice, and insulin digestion within the stomach may be responsible for this failure (Pham et al., 2016). Therefore, large doses of insulin may be required in humans, and it has been reported that a high-dose oral insulin (67.5 mg daily) induced an immune response in genetically at-risk healthy children, which may be protective for T1DM, and no adverse event, such as hypoglycemia, was detected (Bonifacio et al., 2015). To improve the antigen delivery efficiency, delivery carriers that could protect the administered antigens from degradation occurring during the gastrointestinal tract (GIT) transit may be needed. Among the reported oral tolerance induction studies in T1DM, various vehicles have been applied to deliver disease-associated autoantigens, such as transgenic plants and recombinant lactic acid bacteria (LAB) (Mao et al., 2020).

Obtained from natural LAB, bacterial-like particles (BLPs) exhibit a higher binding ability toward fusion proteins containing the corresponding anchor domain and less anticarrier response, compared to live LAB cells (Saluja et al., 2010; Steen et al., 2003). The predominant application of the surface-engineered BLPs displaying the related proteins or peptides is vaccine or immunotherapy (Mao et al., 2016). In our previous studies, two monovalent BLPs vaccines, which contain a single-chain insulin (SCI-59) analog (BLPs-SCI-59) (Mao et al., 2019; Mao et al., 2017) and the intracellular domain of insulinoma-associated protein 2 (IA-2ic) (BLPs-IA-2ic) (Mao et al., 2022), respectively, have been successfully constructed. Compared to the free fusion proteins, fusion proteins bound to BLPs exhibit higher stability in vitro. Compared to the empty BLPs and free proteins, oral vaccination with both monovalent BLPs vaccines could significantly suppress the onset of T1DM in NOD mice by stimulating the corresponding antigen-specific tolerance, respectively (Mao et al., 2019; Mao et al., 2022). Based on these previous studies about the construction and in vivo evaluation of such monovalent BLPs vaccine (BLPs-SCI-59 or BLPs-IA-2ic), our present study aimed to construct two bivalent BLPs vaccines which simultaneously deliver SCI-59 and IA-2ic, and evaluate whether there is an additive beneficial effect on tolerance induction and prevention of T1DM by using bivalent BLPs vaccines.

## Materials and methods

### Mice

NOD female 4-week-old mice (NOD/LtJ) were purchased from the Shanghai Laboratory Animal Company (SLAC, Shanghai, China) and kept in a pathogen-free condition with light (12-h light/12-h dark cycle) and temperature of (23 ± 1 °C) control. Animals had free access to standard mouse chow as well as sterilized drinking water. The animal’s care and all experimental procedures in this study were performed in compliance with the Chinese Experimental Animals Administration Legislation and approved by the Animal Experimental Ethics Committee of Huaiyin Normal University (Ethical approval number: 20180312-2).

### Production of BLPs vaccines

As described previously, two monovalent BLPs vaccines, including BLPs-SCI-59 (Mao et al., 2019, 2017) and BLPs-IA-2ic (Mao et al., 2022), were obtained by adding 3.75 × 10^9^ MG1363 BLPs (2.5 × 10^10^ BLPs/ml) to the corresponding fusion protein suspensions (SCI-59-3LysM or IA-2ic-3LysM). The collected BLPs vaccines were washed and resuspended in 150 μl sterile phosphate-buffered saline (PBS, pH 7.0), respectively. Two types of bivalent BLPs vaccine were prepared. The first one (Bivalent vaccine-mix) was obtained by mixing 150 μl BLPs-SCI-59 vaccine with 150 μl BLPs-IA-2ic vaccine. The other one (Bivalent vaccine-SA), which refers to the simultaneous attachment of both SCI-59 and IA-2ic to the same bacterium-like particle, was prepared by adding 7.5 × 10^9^ MG1363 BLPs to the fusion protein suspension containing the corresponding amount of both SCI-59-3LysM and IA-2ic-3LysM. When the binding reaction ended, the collected particles were washed and resuspended in 300 μl PBS. Both monovalent and bivalent BLPs vaccines contained an equal amount of the corresponding antigens (SCI-59 or IA-2ic). In order to confirm the simultaneous binding of both antigens to the same particle, anti-IA-2 antibody followed by Alexa Fluor 488-labeled secondary antibody, and anti-insulin antibody followed by Alexa Fluor 594-labeled secondary antibody were used in immunofluorescence microscopy analysis as described previously (Mao et al., 2017). All antibodies used in this study were obtained from Sangon Biotech (Shanghai, China) unless otherwise specified.

### Vaccination and estimate of disease

Ninety NOD mice were randomly divided into six groups (*n* = 15 mice/group), including PBS group, BLPs group, BLPs-SCI-59 group, BLPs-IA-2ic group, Bivalent vaccine-mix group, and Bivalent vaccine-SA group. Mice in PBS group and BLPs group were fed 300 μl PBS and 300 μl BLPs (BLPs-3LysM) by oral gavage, respectively. Mice in BLPs-SCI-59 group were fed a mixture (300 μl) containing 150 μl BLPs-SCI-59 vaccine and 150 μl BLPs (BLPs-3LysM). For BLPs-IA-2ic group, mice were fed a mixture (300 μl) containing 150 μl BLPs-IA-2ic vaccine and 150 μl BLPs (BLPs-3LysM). Mice in Bivalent vaccine-mix group were fed 300 μl Bivalent vaccine-mix vaccine containing 150 μl BLPs-SCI-59 vaccine and 150 μl BLPs-IA-2ic vaccine. Mice in Bivalent vaccine-SA group were fed 300 μl Bivalent vaccine-SA vaccine containing the same amount of SCI-59 and IA-2ic as the Bivalent vaccine-mix group. For gavage experiments (beginning at five weeks of age), mice in each group were gavaged with the corresponding agent once a day within the first seven days, and then three times every seven days until the gavage ended at age 20 weeks. Beginning at 15 weeks of age, mouse serum samples were collected weekly and stored at −80 °C until further analysis. Body weight and blood glucose level were evaluated weekly. Combined with the symptoms, including polyuria and weight loss, animals could be considered to be diabetic when the blood glucose level is above 11 mmol/L for two successive weeks. Morbidity and mortality were recorded until 40 weeks of age.

### Serum antibody analysis

Antigen-specific antibodies in mouse serum were analyzed by ELISA after the last dose at 20 weeks of age. Using purified SCI-59 or IA-2 protein as the well-coating antigen, serially diluted serum samples from all mice were added and incubated. HRP-labeled secondary antibodies used include anti-mouse IgG, IgG1, and IgG2a. Using a microplate reader (Bio-Rad, CA, USA), OD_450_ was measured and used to calculate the corresponding antibody titer as defined previously (Liu et al., 2016).

### Oral glucose tolerance test

Oral glucose tolerance test (OGTT) was performed in non-diabetic mice in each group at 21 weeks of age (*n* = 13 in PBS group; *n* = 13 in BLPs group; *n* = 14 in BLPs-SCI-59 group; *n* = 15 in BLPs-IA-2ic group; *n* = 15 in Bivalent vaccine-mix group; *n* = 15 in Bivalent vaccine-SA group). Blood samples were collected after an overnight fast, and then applied to the measurement of basal blood glucose levels. Then, each mouse received a glucose solution (2 g/kg) intraperitoneally. Blood glucose level of each mouse was measured and recorded at 0, 15, 30, 45, 60, 75, 90, 105, and 120 min after injection.

### Analysis of serum insulin and cytokine

At 40 weeks of age, serum insulin levels were measured with the help of a commercial ELISA kit (Sangon Biotech, Shanghai, China). For mice still alive at 40 weeks of age, serum samples were collected and analyzed before euthanization. For mice that died during the observation period, serum samples collected at last week before death were analyzed. At the same time, levels of interleukin (IL)-2, IL-4, IL-10, and interferon (IFN) -γ were analyzed in serum with the help of the corresponding ELISA kit (Sangon Biotech, Shanghai, China). The procedure of ELISA was conducted following the manufacturer’s instructions.

### Pancreas histopathology

In order to evaluate the potential protective effect on the pancreas provided by different treatments, surviving mice (40-week-old) in all groups (*n* = 5 in PBS group; *n* = 4 in BLPs group; *n* = 9 in BLPs-SCI-59 group; *n* = 9 in BLPs-IA-2ic group; *n* = 15 in Bivalent vaccine-mix group; *n* = 13 in Bivalent vaccine-SA group) were euthanized, and pancreas samples were harvested and applied to insulitis investigation as described previously (Lang et al., 2017). For each animal, at least 20 islets were analyzed.

### Splenocyte proliferation

Splenocytes isolated from the above-euthanized mice were suspended and seeded in 96-well plates (1 × 10^6^ cells per well), and then stimulated with medium, BSA (final concentration: 10 μg/ml), concanavalin A (ConA, final concentration: 10 μg/ml, Sangon Biotech, Shanghai, China), purified SCI-59 (final concentration: 10 μg/ml) or IA-2 protein (final concentration: 10 μg/ml, Sangon Biotech, Shanghai, China), respectively. Cell proliferation was measured by WST-8 test (Beyotime, Shanghai, China) after a 72-h incubation, and then the corresponding stimulation index (SI) could be determined as defined previously (Mao et al., 2022).

**Figure 1.**
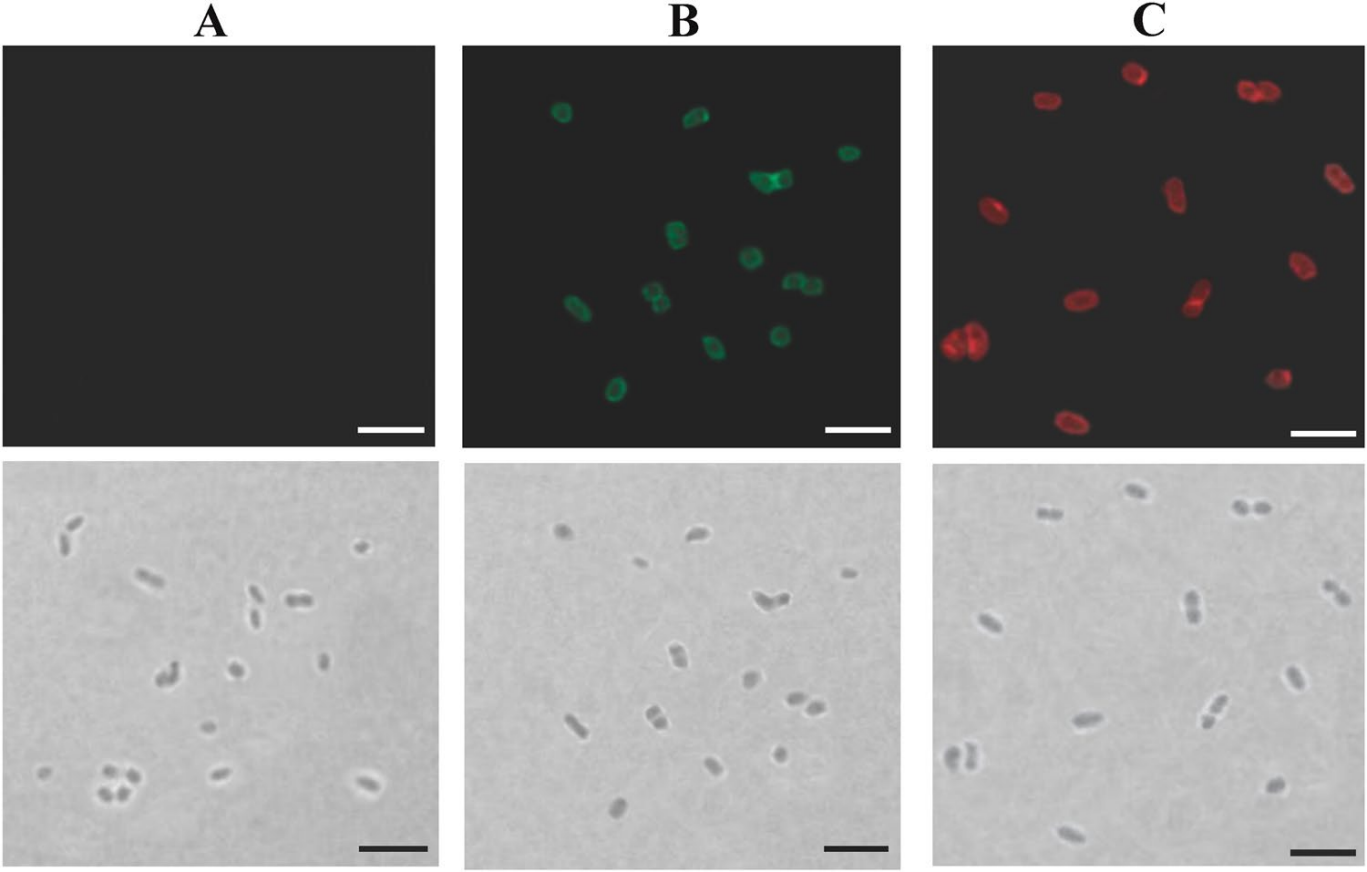
Analysis of bivalent BLPs vaccine binding IA-2ic and SCI-59 simultaneously (Bivalent vaccine-SA) by immunofluorescence microscopy. Immunofluorescence (top) and bright-field (bottom) microscopy images of BLPs alone (A) and bivalent BLPs vaccine (B and C). Two equal parts were taken from the obtained bivalent BLPs vaccine. One was revealed by incubation with an anti-IA-2 antibody followed by Alexa Fluor 488-labeled secondary antibody (green, B) and the other one was revealed by incubation with an anti-insulin antibody followed by Alexa Fluor 594-labeled secondary antibody (red, C). A similar analysis was performed for BLPs alone, and no fluorescence signal (green or red) was detected. Scale bar: 5 μm.

### ELISPOT

Mouse IFN-γ and IL-4 ELISPOT kits (U-CyTech, Utrecht, the Netherlands) were used to detect T cells producing the corresponding cytokine. As described above, isolated splenocytes (1 × 10^6^ cells per well) were cultured and stimulated with medium, SCI-59 or IA-2. After a 72-h incubation, cells were transferred into an antibody-coated 96-well ELISPOT plate, serially diluted, and incubated for another 24 h. The procedure of ELISPOT was performed per the manufacturer’s protocols, and the spots were counted manually under a dissection microscope. The final numbers of cells producing the corresponding cytokine per 10^6^ splenic cells were calculated by subtracting the background spots presented in medium-stimulated samples.

### Tregs analysis

Lymphocytes were isolated from the euthanized mice’s pancreatic lymph nodes (PLNs) using a commercial mouse lymphocyte isolation kit provided by Beijing Solarbio Science & Technology, China. The mouse CD4^+^CD25^+^Foxp3^+^ Tregs staining kit was used to stain the collected lymphocytes following the manufacturer’s recommendations (Invitrogen, CA, USA). After washing, cells were analyzed on Accuri C6 plus flow cytometer (BD Biosciences, Franklin Lakes, NJ, USA).

### Statistical analysis

Data were analyzed with the help of Graphpad Prism 6 software (La Jolla, CA, USA). For comparative analysis of T1DM incidence curves and survival curves, the log-rank (Mantel-Cox) test was applied. Data are expressed as means ± SD. For multiple comparisons, the independent-samples t-test or ANOVA was used. *p* < 0.05 was considered statistically significant.

## Results

### Production of BLPs vaccines

For monovalent BLPs vaccines (BLPs-SCI-59 or BLPs-IA-2ic), 3.75 × 10^9^ MG1363 BLPs were added to the fusion protein suspensions containing the same amount of SCI-59-3LysM (Mao et al., 2019) or IA-2ic-3LysM (Mao et al., 2022) as described previously, and all these fusion proteins successfully bound to the BLPs (data not shown). A Bivalent vaccine-mix was obtained by mixing an equal amount (150 μl) of each monovalent BLPs vaccine produced as described above. For Bivalent vaccine-SA, 7.5 × 10^9^ MG1363 BLPs were added to the fusion protein suspension mixtures containing the same amount of both SCI-59-3LysM and IA-2ic-3LysM used in producing monovalent BLPs vaccines. After binding, the supernatant contained no free fusion protein, and therefore all these fusion proteins could bind to the BLPs successfully (data not shown). As shown in Figure 1, Bivalent vaccine-SA simultaneously displaying SCI-59 and IA-2ic was verified by immunofluorescence microscopy.

### Oral administration of BLPs vaccines prevented T1DM

**Figure 2.**
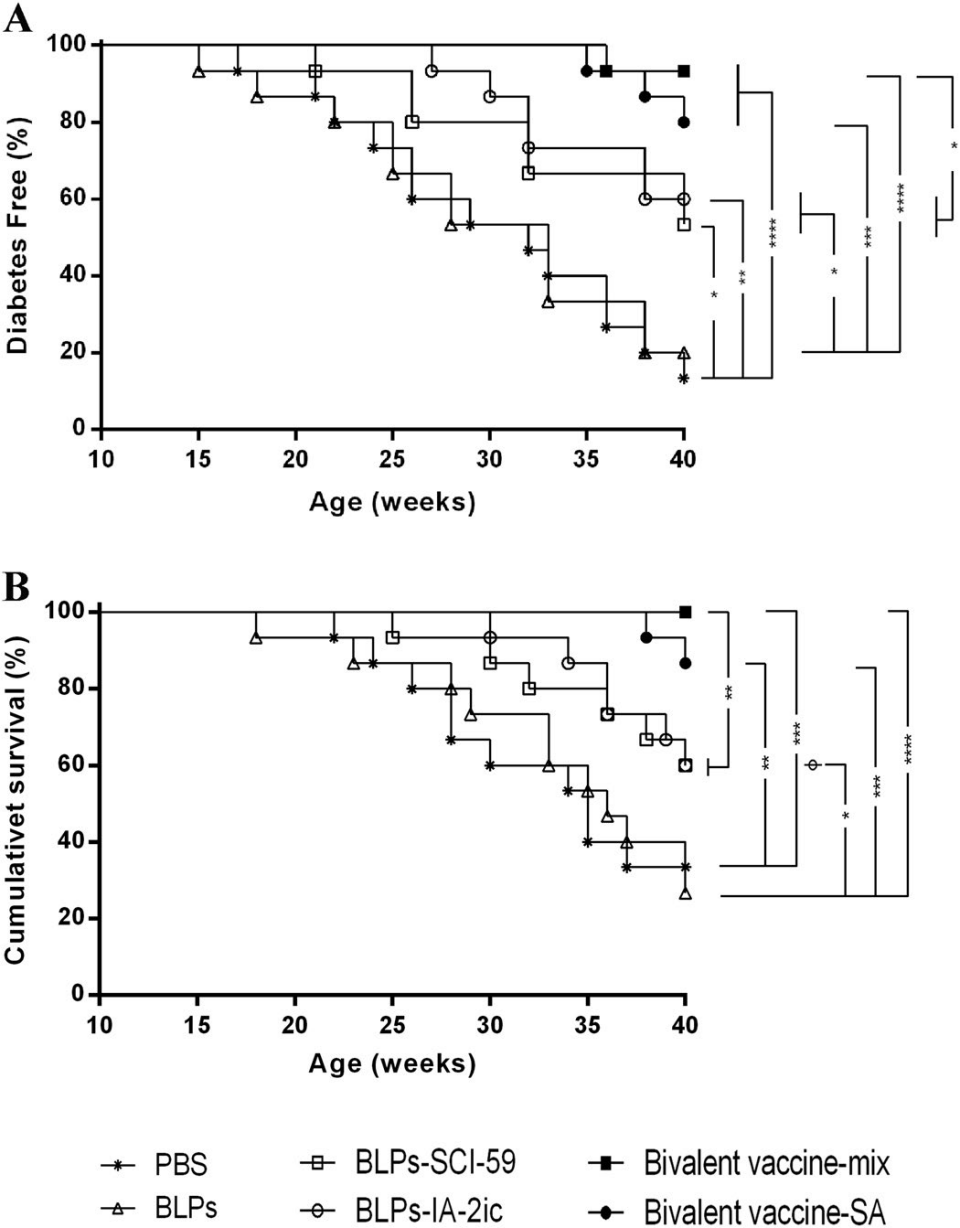
Effect of different treatments on T1DM in NOD mice. Mice (*n* = 15 per group) were fed PBS, BLPs, BLPs-SCI-59, BLPs-IA-2ic, Bivalent vaccine-mix, and Bivalent vaccine-SA once daily during the first week and then three times per week in the following 15 weeks. The development of diabetes was monitored until 40 weeks of age by observing the onset of hyperglycemia and the related symptoms. (A) The frequency of diabetes-free mice over time. (B) Survival curve of NOD mice. *p < 0.05, **p < 0.01, ***p < 0.001, ****p < 0.0001.

Beginning at five weeks of age (prior to diabetes onset), NOD mice were treated with PBS, BLPs, BLPs-SCI-59, BLPs-IA-2ic, Bivalent vaccine-mix, or Bivalent vaccine-SA. During the observation period (5-40 weeks old), morbidity and mortality were monitored and analyzed (Figure 2). Mice treated with PBS and BLPs began to develop T1DM at 17 and 15 weeks, respectively. In the PBS group and BLPs group, the corresponding disease incidence (40-week-old) was 87% (13/15) and 80% (12/15), and the survival rate (40-week-old) was 33% (5/15) and 27% (4/15), respectively. Compared to the PBS group, the BLPs group exhibited no significant difference in the disease incidence and survival rate. Therefore, treatment with BLPs (BLPs-3LysM) did not affect the development of diabetes. Mice treated with BLPs-SCI-59, BLPs-IA-2ic, Bivalent vaccine-mix, and Bivalent vaccine-SA began to develop T1DM at 21, 27, 36, and 35 weeks, respectively. Therefore, diabetes onset was delayed among groups receiving various BLPs vaccines treatment, especially in mice treated with bivalent BLPs vaccines, as compared to the PBS group and BLPs group. At 40 weeks of age, the incidence of diabetes was 47% (7/15, BLPs-SCI-59 group), 40% (6/15, BLPs-IA-2ic group), 7% (1/15, Bivalent vaccine-mix group), and 20% (3/15, Bivalent vaccine-SA group), and the survival rate was 60% (9/15, BLPs-SCI-59 group), 60% (9/15, BLPs-IA-2ic group), 100% (15/15, Bivalent vaccine-mix group) and 87% (13/15, Bivalent vaccine-SA group). As shown in Figure 2(A), the incidence of T1DM reduced significantly in the BLPs vaccines-treated groups compared to those treated with PBS or BLPs. Treatment with BLPs-SCI-59 and BLPs-IA-2ic showed no significant difference in disease incidence. Similarly, there was no significant difference in disease incidence between the Bivalent vaccine-mix group and Bivalent vaccine-SA group. However, the incidence of diabetes in mice treated with Bivalent vaccine-mix decreased significantly, as compared with that of the monovalent BLPs vaccines-treated mice. As shown in Figure 2(B), the mice treated with bivalent BLPs vaccines (Bivalent vaccine-mix group and Bivalent vaccine-SA group) exhibited a significantly higher survival rate than those treated with PBS and BLPs. Treatment with two monovalent BLPs vaccines had no significant survival difference, and similarly, no obvious survival difference was seen between the Bivalent vaccine-mix group and Bivalent vaccine-SA group. Notably, the Bivalent vaccine-mix group exhibited a higher survival rate than those treated with two monovalent BLPs vaccines. Therefore, these data indicate that oral vaccination with these BLPs vaccines prevented T1DM in NOD mice. In addition, and importantly, oral administration of Bivalent vaccine-mix shows to be more effective for the suppression of T1DM than the monovalent BLPs vaccines-treatment.

### Oral administration of BLPs vaccines enhanced glucose tolerance

**Figure 3.**
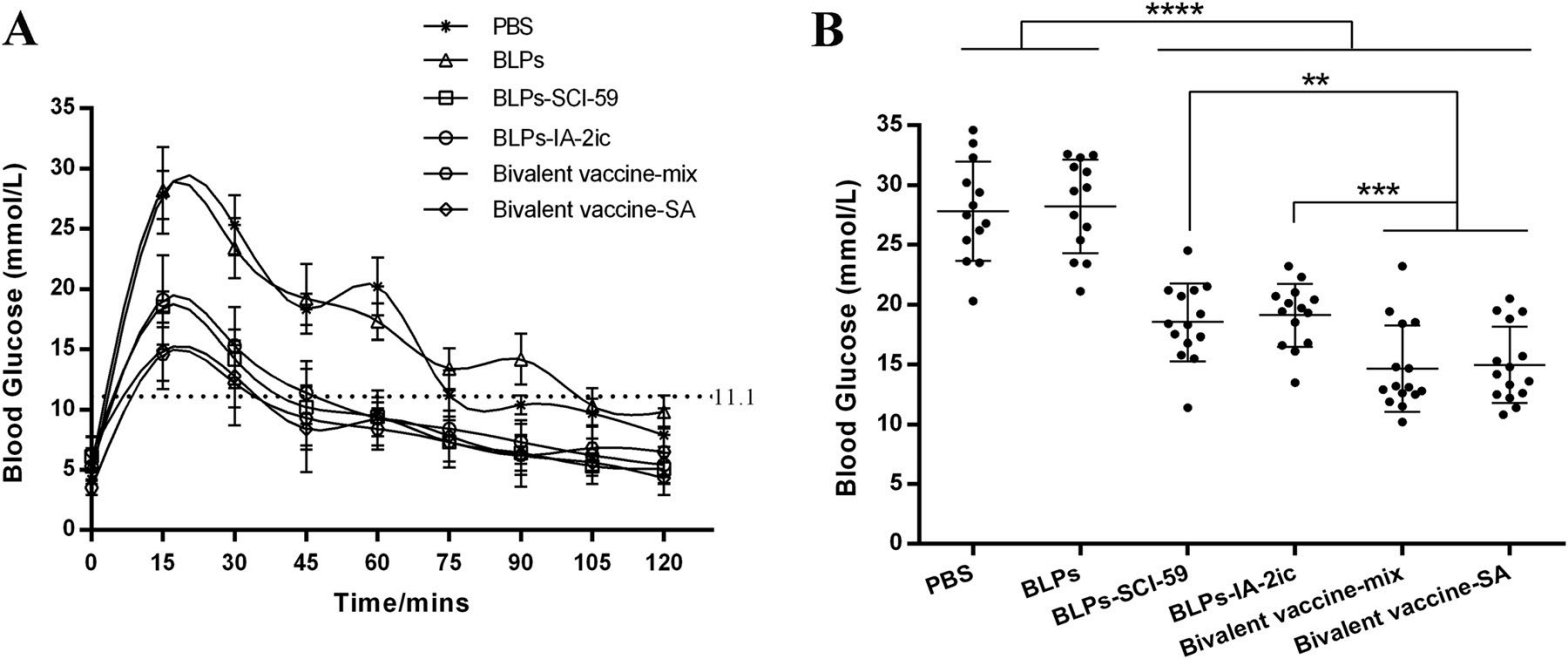
Effect of different treatments on glucose tolerance in NOD mice. At 21 weeks of age, non-diabetic mice in each group (*n* = 13 in PBS group; *n* = 13 in BLPs group; *n* = 14 in BLPs-SCI-59 group; *n* = 15 in BLPs-IA-2ic group; *n* = 15 in Bivalent vaccine-mix group; *n* = 15 in Bivalent vaccine-SA group) were selected for glucose tolerance test. (A) Blood glucose was measured at selected time points after glucose injection. (B) Comparative analysis of blood glucose levels among these groups at 15 min. Data are shown as means ± SD. **p < 0.01, ***p < 0.001, ****p < 0.0001.

To further assess the potential preventive effect of different BLPs vaccines against T1DM, an OGTT was conducted in non-diabetic mice in all groups at 21 weeks of age (Figure 3). After an overnight fast, all mice exhibited a similar blood glucose level. After glucose injection, all the mice’s blood glucose levels rose and reached the corresponding peak levels at 20 min, and then decreased (Figure 3A). Within the first 20 min, the blood glucose levels of mice in the PBS and BLPs group increased rapidly. However, the BLPs vaccines-treated groups (especially the bivalent BLPs vaccines-treated groups) exhibited relatively gentle increasing patterns (Figure 3A). As shown in Figure 3(B), a significant reduction in blood glucose levels measured at 15 min after glucose injection was observed in the groups receiving BLPs vaccines treatment compared to the PBS and BLPs groups. In addition, significant differences in the levels of blood glucose measured at 15 min after glucose injection were observed between the monovalent BLPs vaccines-treated groups and the bivalent BLPs vaccines-treated groups. After reaching peak values, the PBS and BLPs group returned to normal levels at approximately 80 min and 105 min, respectively. However, the BLPs vaccines-treated groups spent less time returning to normal blood sugar levels as all these groups returned to normal at approximately 45 min (Figure 3A). In addition, the blood glucose fluctuation was relatively larger in the PBS and BLPs group than in the BLPs vaccines-treated groups (Figure 3A). These results indicated that NOD mice treated with the BLPs vaccines (especially the bivalent BLPs vaccines-treated groups) showed enhanced glucose tolerance.

### Oral administration of BLPs vaccines reduced insulitis and preserved insulin secretion

**Figure 4.**
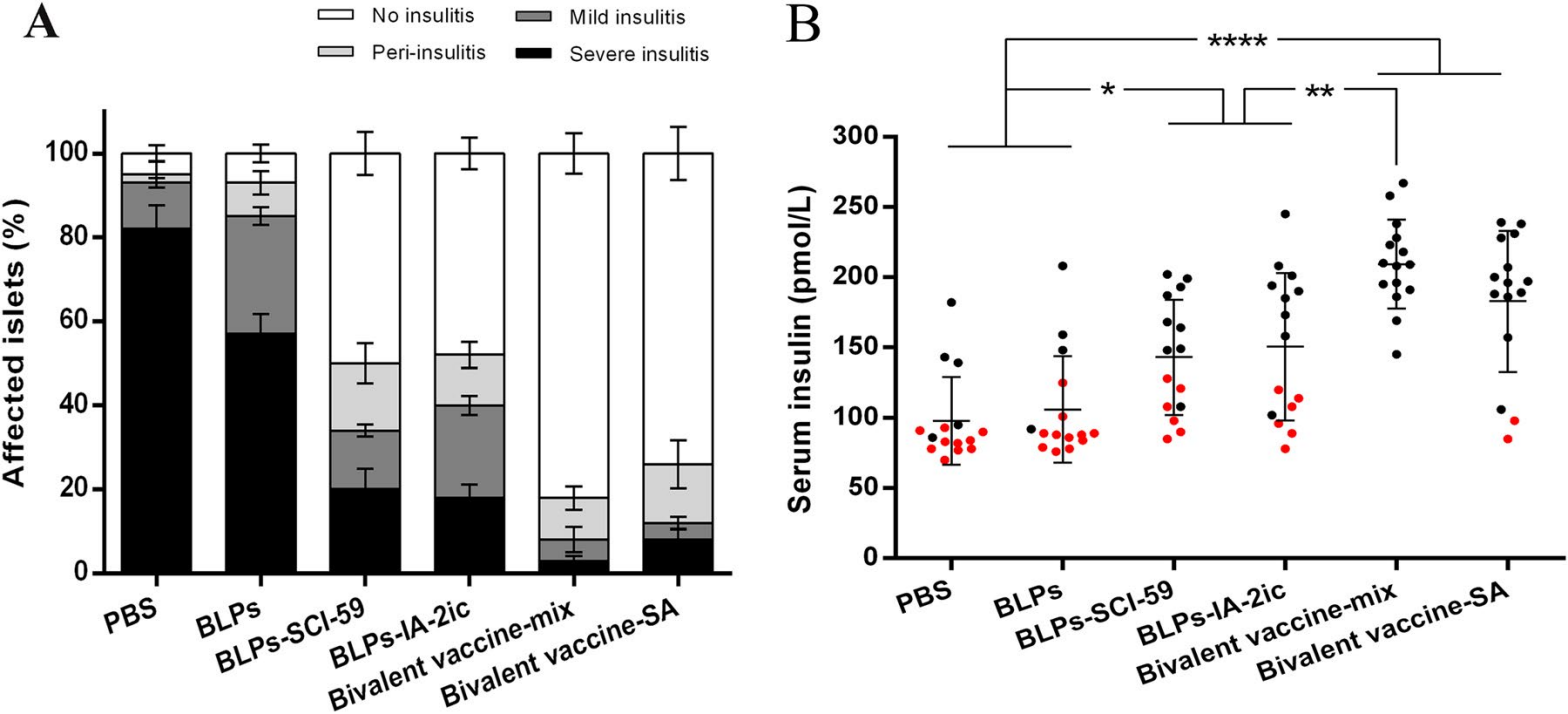
Effect of different treatments on insulitis and preservation of insulin secretion in NOD mice. (A) Frequency of islets with various grades of insulitis. At the end of the observation period, pancreas samples were obtained from surviving mice in each group (*n* = 5 in PBS group; *n* = 4 in BLPs group; *n* = 9 in BLPs-SCI-59 group; *n* = 9 in BLPs-IA-2ic group; *n* = 15 in Bivalent vaccine-mix group; *n* = 13 in Bivalent vaccine-SA group) for histological examination. At least 20 islets per mouse were analyzed. (B) Serum insulin levels of NOD mice (*n* = 15 per group). For the mice still alive at the end of the observation period (black dots), the serum insulin levels at 40 weeks of age are recorded and analyzed. For the mice that died during the observation period (red dots), the serum insulin levels at the last week before death are recorded and analyzed. Data are shown as means ± SD. * *p* < 0.05, ** *p* < 0.01, **** *p* < 0.0001.

In order to determine the mechanism accounting for T1DM prevention by BLPs vaccines, the progression of insulitis in animals was evaluated and analyzed at 40 weeks of age. As shown in Figure 4(A), the BLPs vaccines-treated groups exhibited a greater percentage of intact islets (without insulitis) compared to the PBS and BLPs group. In addition, the percentage of intact islets in the bivalent BLPs vaccines-treated groups (Bivalent vaccine-mix group: 92%; Bivalent vaccine-SA group: 88%) was higher than that in the monovalent BLPs vaccines-treated groups (BLPs-SCI-59 group: 66%; BLPs-IA-2ic group: 60%). In addition, serum insulin levels were significantly higher among the groups receiving BLPs vaccines treatment, as compared to the PBS and BLPs group (Figure 4B). Especially, the Bivalent vaccine-mix group exhibited a significant higher serum insulin level than the monovalent BLPs vaccines-treated groups. Therefore, it is suggested that oral vaccination with these BLPs vaccines (especially the Bivalent vaccine-mix) preserved islet integrity and maintained their ability to secrete insulin.

### Oral administration of BLPs vaccines activated antigen-specific Th2-type immune response

**Figure 5.**
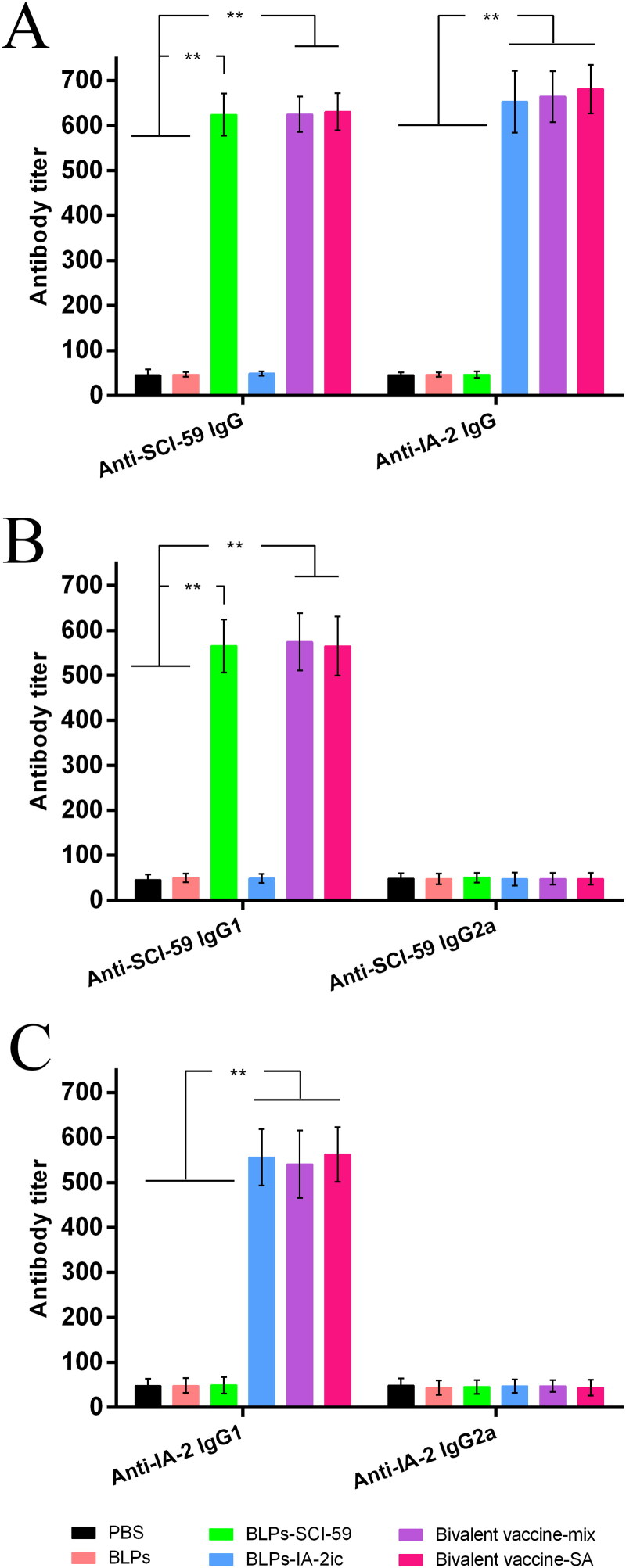
Analysis of serum antigen-specific antibodies among different treatment groups. At 20 weeks of age, serum from all mice (*n* = 15 per group) was quantified for anti-SCI-59 or anti-IA-2 antibodies and their subtypes, respectively. Data are shown as means ± SD. ** *p* < 0.01.

At 20 weeks of age, serum antigen-specific IgG levels were evaluated in all NOD mice. It has been shown that oral administration of the free SCI-59 (Mao et al., 2019) or free IA-2ic-3LysM (Mao et al., 2022) could not induce the production of the corresponding serum antigen-specific IgG, and this may be due to the digestion of the orally-administered proteins occurred during passage through the GIT. Unlike the orally-administered free antigens, antigens delivered by BLPs (BLPs vaccines) induced significantly higher levels of antigen-specific serum IgG (anti-SCI-59 IgG and/or anti-IA-2 IgG) in NOD mice (Figure 5A). No obvious difference in the corresponding antigen-specific IgG levels was observed between the monovalent BLPs vaccines-treated groups and the bivalent BLPs vaccines-treated groups. In addition, the type of the bivalent BLPs vaccines did not influence the induction of antigen-specific serum IgG (Figure 5A). These results suggested that BLPs vaccines could effectively deliver the corresponding antigens to the gut immune system, inducing an antigen-specific humoral immune response.

Additionally, antigen-specific IgG1 (Th2-dependent isotype) and IgG2a (Th1-dependent isotype) levels in serum were measured. As shown in Figure 5(B), the anti-SCI-59 antibodies in groups receiving SCI-59 treatment were mainly IgG1 subclass. Similar results of the anti-IA-2 antibodies in groups receiving IA-2 treatment were observed (Figure 5C). Collectively, it is suggested that treatment with these BLPs vaccines could induce antigen-specific Th2-type, but not Th1-type, immune response. Especially, mice immunized with the bivalent BLPs vaccines exhibited both SCI-59- and IA-2-specific Th2 response.

### Oral administration of BLPs vaccines specifically inhibited T cell proliferation

**Figure 6.**
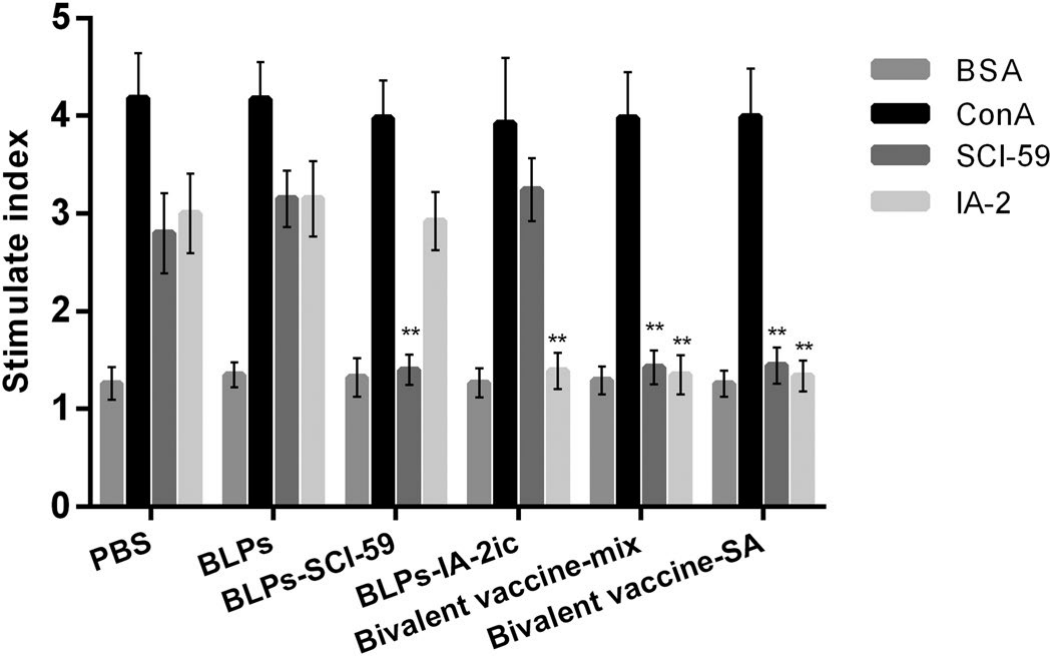
Effect of different treatments on T cell proliferation. At the end of the observation period (40-week-old), all alive mice in each group (*n* = 5 in PBS group; *n* = 4 in BLPs group; *n* = 9 in BLPs-SCI-59 group; *n* = 9 in BLPs-IA-2ic group; *n* = 15 in Bivalent vaccine-mix group; *n* = 13 in Bivalent vaccine-SA group) were euthanized and subjected to splenocyte proliferation test by simulating with BSA, ConA, SCI-59, and IA-2. For each mouse, at least three independent experiments were performed. Data are shown as means ± SD. ** *p* < 0.01.

Splenocytes isolated from all surviving mice at 40 weeks of age were applied to the proliferation assay to evaluate whether antigen-specific tolerance was successfully stimulated. As shown in Figure 6, all mice exhibited no response to BSA (negative control), however, all mice exhibited similar strong proliferative responses to ConA (positive control), which indicated that oral administration of BLPs vaccines did not influence the general reactivity of T cell. Upon stimulation with SCI-59 or IA-2, the proliferation of splenocytes varied among groups (Figure 6). Splenocytes from the PBS and BLPs group exhibited spontaneous reactivity to SCI-59 and IA-2. Splenocytes from BLPs-SCI-59 group and BLPs-IA-2ic group showed a significantly reduced proliferative response to SCI-59 and IA-2, respectively. However, BLPs-SCI-59 group exhibited spontaneous reactivity to IA-2, and BLPs-IA-2ic group exhibited spontaneous reactivity to SCI-59. In contrast, the bivalent BLPs vaccines-treated groups showed a significantly reduced SI to both SCI-59 and IA-2 as compared to the PBS and BLPs groups (Figure 6). No obvious difference was seen between the Bivalent vaccine-mix group and the Bivalent vaccine-SA group. Therefore, it is suggested that oral vaccination with these BLPs vaccines could downregulate the spontaneous T cell proliferation response to the corresponding antigen. Especially, treatment with the bivalent BLPs vaccines induced immune tolerance to both SCI-59 and IA-2, consequently suppressing disease development.

### Oral administration of BLPs vaccines induced a Th1 to Th2 shift

**Figure 7.**
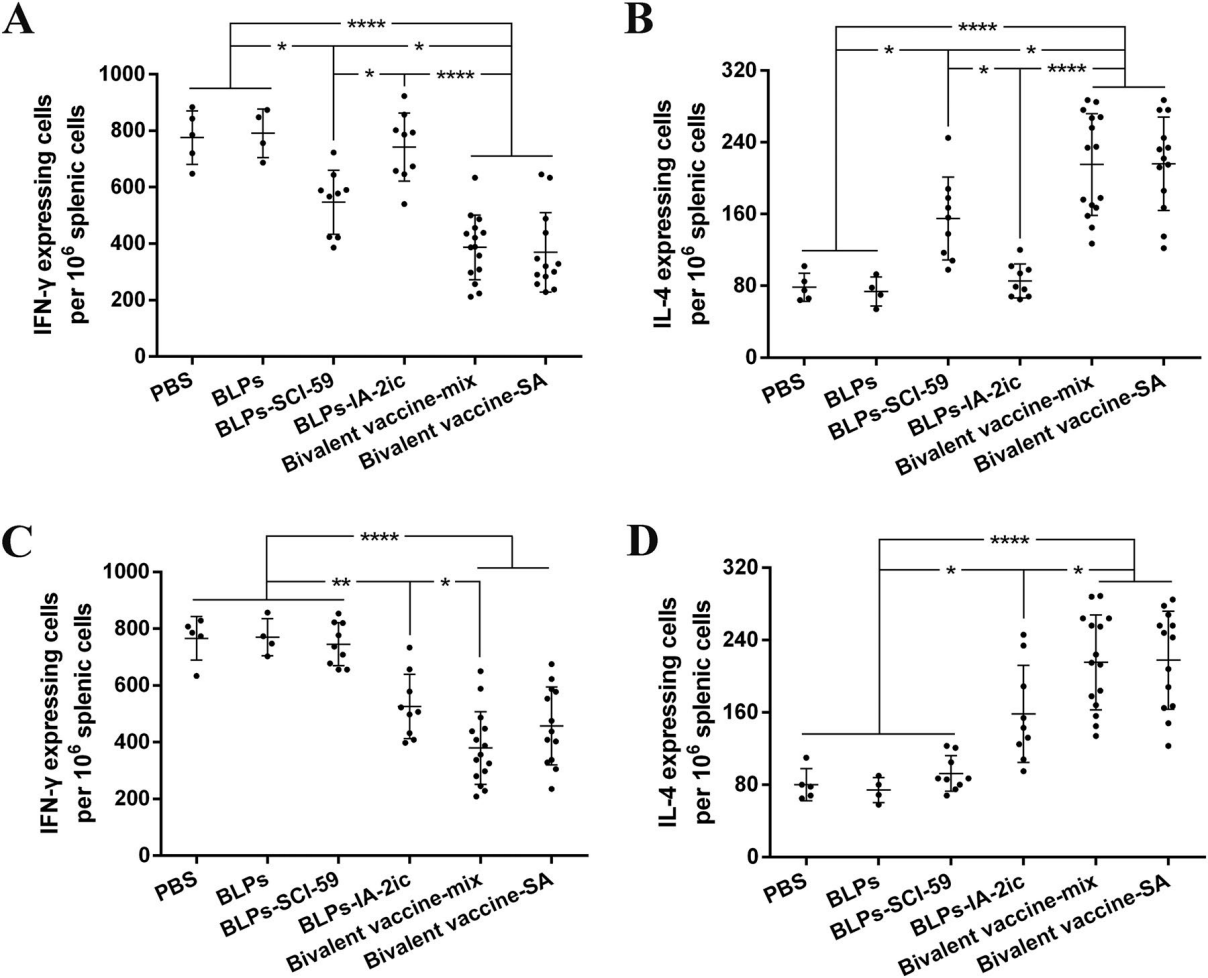
ELISPOT analysis of IL-4/IFN-γ-producing T cells. At the end of the observation period (40-week-old), all alive mice in each group (*n* = 5 in PBS group, *n* = 4 in BLPs group, *n* = 9 in BLPs-SCI-59 group, *n* = 9 in BLPs-IA-2ic group, *n* = 15 in Bivalent vaccine-mix group, *n* = 13 in Bivalent vaccine-SA group) were euthanized, and the collected splenocytes (10^6^/well) were stimulated with SCI-59 (A and B) or IA-2 (C and D). T cells producing IFN-γ (A and C) or IL-4 (B and D) were detected. For each mouse, data are representative of at least three independent experiments performed at least three times. For dot plots, each dot represents one mouse. Data are shown as means ± SD. * *p* < 0.05, ** *p* < 0.01, **** *p* < 0.0001.

At 40 weeks of age, all surviving mice’s splenocytes were cultured with SCI-59 and IA-2, respectively, and the frequency of T cells secreting IFN-γ (Th1) or IL-4 (Th2) was investigated. Upon stimulation with SCI-59 (Figure 7A and B), it was observed that Th2 cells significantly increased (Figure 7B) and Th1 cells significantly reduced (Figure 7A) in groups receiving SCI-59 treatment compared with groups without SCI-59 treatment. Upon stimulation with IA-2 (Figure 7C and D), a similar change in Th2 cells (Figure 7D) and Th1 cells (Figure 7C) was observed in groups receiving IA-2 treatment compared with groups without IA-2 treatment. Importantly, in most cases, a significant increase in Th2 cells (Figure 7B and D) and a significant reduction in Th1 cells (Figure 7A and C) was observed in the bivalent BLPs vaccines-treated groups compared with the corresponding monovalent BLPs vaccines-treated groups. It is suggested that treatment with these BLPs vaccines (especially the bivalent BLPs vaccines) could induce Th2 cells and suppress Th1 cells.

**Figure 8.**
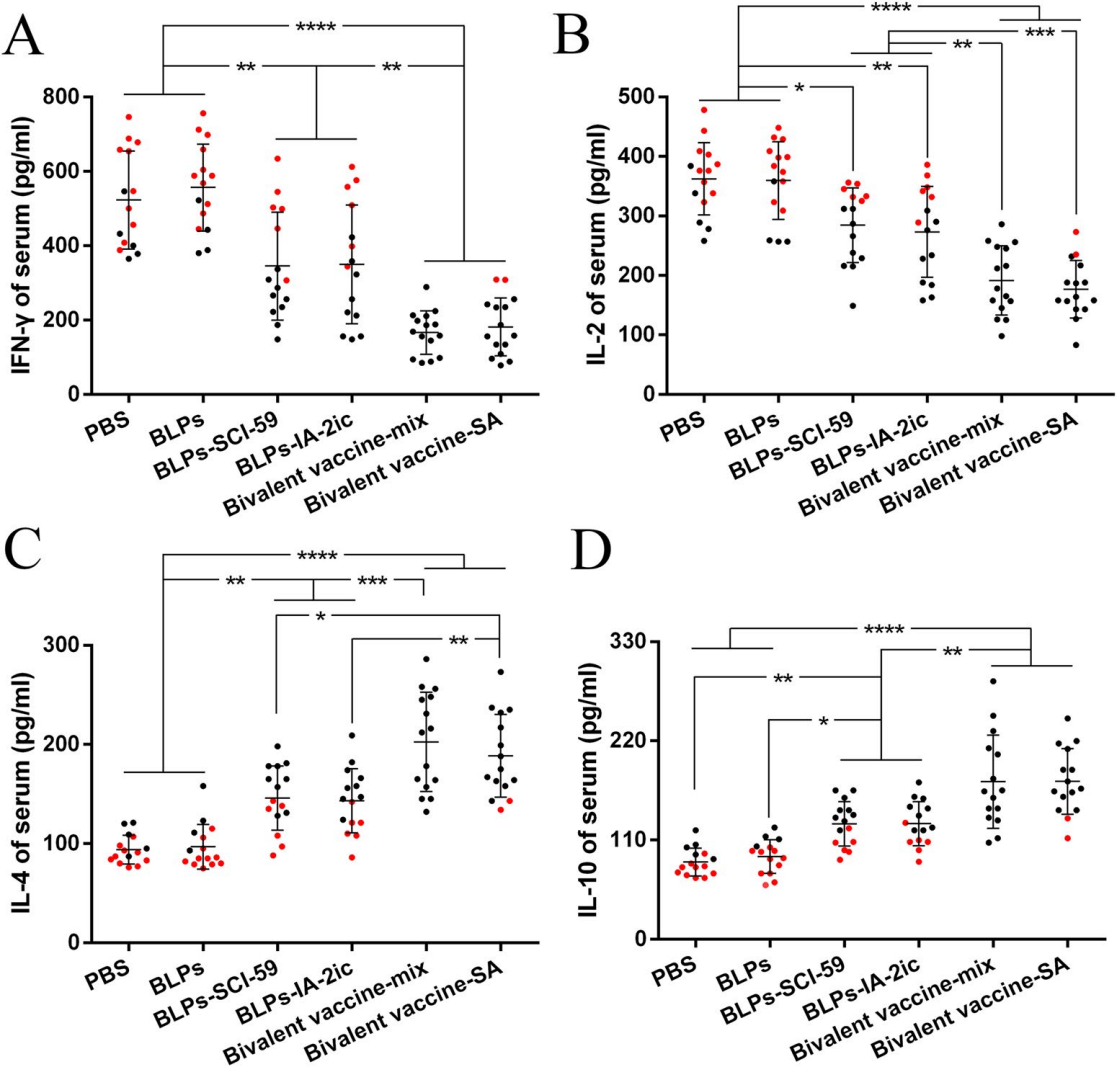
Analysis of serum cytokines among different treatment groups. At the end of the observation period, serum from all mice (*n* = 15 per group) was quantified for IFN-γ (A), IL-2 (B), IL-4 (C), and IL-10 (D), respectively. For the mice still alive at the end of the observation period (black dots), the serum levels of cytokines at 40 weeks of age are recorded and analyzed. For the mice that died during the observation period (red dots), the serum levels of cytokines at the last week before death are recorded and analyzed. For each mouse, data are representative of at least three independent experiments performed at least three times. For dot plots, each dot represents one mouse. Data are shown as means ± SD. * *p* < 0.05, ** *p* < 0.01, *** *p* < 0.001, **** *p* < 0.0001.

In addition, the serum levels of IL-2 and IFN-γ (Th1 cytokines) and IL-4 and IL-10 (Th2 cytokines) were measured and analyzed. As shown in Figure 8, treatment with BLPs vaccines significantly decreased IFN-γ levels (Figure 8A) and IL-2 levels (Figure 8B) compared with the PBS and BLPs group. Conversely, treatment with BLPs vaccines significantly enhanced IL-4 levels (Figure 8C) and IL-10 levels (Figure 8D) compared with the PBS and BLPs group. Importantly, treatment with the bivalent BLPs vaccines led to a stronger suppression of Th1 cytokines (Figure 8 A and B) and a stronger induction of Th2 cytokines (Figure 8 C and D) than that of treatment with the monovalent BLPs vaccines. The enhanced Th2 cytokines and reduced Th1 cytokines stimulated by BLPs vaccines could further support the above ELISPOT findings. Collectively, it is suggested that oral administration of these BLPs vaccines (especially the bivalent BLPs vaccines) could induce a shift from Th1- to Th2-type immunity.

### Oral administration of BLPs vaccines enhanced Tregs differentiation

**Figure 9.**
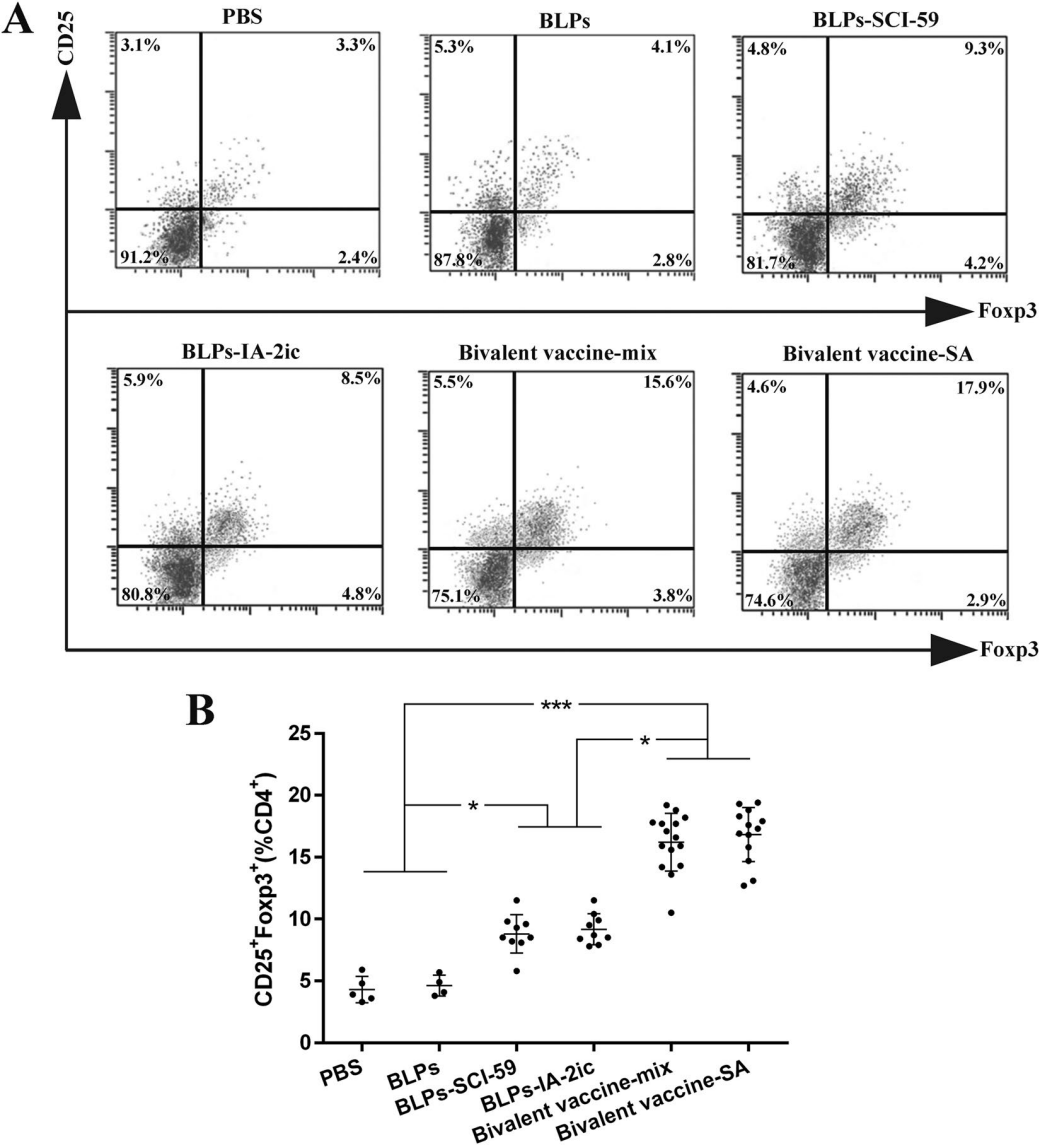
Effect of different treatments on Tregs differentiation. At the end of the observation period (40-week-old), all alive mice in each group (*n* = 5 in PBS group, *n* = 4 in BLPs group, *n* = 9 in BLPs-SCI-59 group, *n* = 9 in BLPs-IA-2ic group, *n* = 15 in Bivalent vaccine-mix group, *n* = 13 in Bivalent vaccine-SA group) were euthanized and subjected to the analysis of the proportion of CD4^+^CD25^+^Foxp3^+^ Tregs in PLNs by flow cytometry (A), and comparison among groups was analyzed (B). For each mouse, at least three independent experiments were performed. Data are shown as means ± SD. * *p* < 0.05, *** *p* < 0.001.

PLNs isolated from the surviving mice in each group were subjected to CD4^+^CD25^+^Foxp3^+^ Tregs measurement and quantification (Figure 9A). Based on the comparative analysis among groups (Figure 9B), the percentage of Tregs increased significantly among the monovalent BLPs vaccines- and the bivalent BLPs vaccines-treated groups compared to the PBS and BLPs groups. Importantly, the percentage of Tregs in the bivalent BLPs vaccines-treated groups was significantly higher compared to the monovalent BLPs vaccines-treated groups. These findings suggested that oral vaccination with BLPs vaccines (especially the bivalent BLPs vaccines) enhanced CD4^+^CD25^+^Foxp3^+^ Tregs differentiation.

## Discussion

Based on the significantly increased understanding of the complex immunopathogenesis related to human T1DM (Richardson & Pugliese, 2021), immune therapy exhibits great potential in treating or preventing T1DM (Smigoc Schweiger, 2022). Immune therapy for T1DM has shifted from immunosuppression to tolerance induction as a result of potential side effects or risks related to immunosuppression, particularly among younger children (Felton, 2021). For tolerance induction in T1DM, oral immunization has been an attractive and simple route of drug administration. Studies aiming to induce antigen-specific immune tolerance to treat or prevent T1DM through oral antigen feeding have been widely performed in NOD mice (Mao et al., 2020). Based on these valuable studies, it has been observed that the dose of administered antigen influences the tolerance-inducing effect, therefore influencing the final treatment effect on T1DM. Due to the digestion of the administered antigen when passing through the GIT, large doses of autoantigens would be needed to achieve tolerance (Pham et al., 2016), and this would limit its clinical application as a result of the potentially high cost of producing these antigens. Therefore, effective delivery vehicles as well as improving the expression levels of these autoantigens should be required.

In recent years, numerous studies have shown that LAB exhibit a potential role in delivering biologically active molecules, and therefore promoting the development of mucosal vaccine (Levit et al., 2022). To date, various plasmid-based gene expression systems for LAB have been established, and this highly expands the uses of LAB as drug delivery vehicles through genetic modification (Kazi et al., 2022). However, using these genetically modified organisms may raise significant public and political concerns. As an original alternative to using these genetically modified LAB, non-living LAB BLPs, derived from simple hot acid pretreatment of natural LAB cells, lose the intracellular components (DNA and cytoplasmic proteins) and most cell wall constituents except the rigid peptidoglycan (PGN) matrix, yielding spherical shaped particles (approximately 1-2 µm in diameter) (Van Braeckel-Budimir et al., 2013). LAB BLPs have been used to mix with the existing vaccine antigens, enabling their mucosal application. However, binding of antigens to the surface of LAB BLPs with the help of the PGN binding domain may be preferred in recombinant subunit vaccines. As a result of the exposure of the PGN of the cell-wall, LAB BLPs exhibit a significantly higher fusion protein containing LysM (lysin motif) domains binding capacity compared with untreated LAB cells (Steen et al., 2003). In addition, multiple fusion proteins containing LysM domains can be bound onto one particle, offering the potential to produce and deliver multivalent BLPs vaccines. Alternatively, monovalent BLPs vaccines could be mixed to facilitate flexible vaccine compositions (van Roosmalen et al., 2006).

In our previous studies (Mao et al., 2019; Mao et al., 2017; Mao et al., 2022), we constructed two monovalent BLPs vaccines, including BLPs-SCI-59 and BLPs-IA-2ic, with the help of prokaryotic expression systems. Compared to the corresponding free antigen, oral administration of the such monovalent vaccine (BLPs-SCI-59 or BLPs-IA-2ic) could promote the induction of tolerance, and therefore significantly preventing the onset of T1DM (Mao et al., 2019, 2022). These results indicated that LAB BLPs could efficiently deliver surface-bound recombinant antigens into the intestinal mucosa, where oral tolerance develops (Wambre & Jeong, 2018; Rezende & Weiner, 2022). In addition, based on the review of oral tolerance studies performed in T1DM, a combination of multiple autoantigens, serving as one of the combination therapy strategies, maybe a reasonable and effective immunotherapy strategy against T1DM (Mao et al., 2020, 2020). Therefore, our present study aimed to investigate further whether there is a complementary or additive effect on the prevention of T1DM by constructing and applying two bivalent BLPs vaccines, including Bivalent vaccine-mix and Bivalent vaccine-SA.

Compared to the control groups (PBS group and BLPs group), treatment with various BLPs vaccines could significantly delay and suppress the onset of T1DM. Importantly, oral administration of Bivalent vaccine-mix could significantly reduce morbidity and mortality in T1DM, compared with the monovalent BLPs vaccines-treatment (Figure 2). Therefore, these data would indicate that the combination of SCI-59 and IA-2ic in the Bivalent vaccine-mix leads to an additive beneficial effect on T1DM prevention. This similar additive effect was observed by oral dosing of silkworm-produced fusion protein CTB-Ins-GAD which contains bi-autoantigens (GAD65 and insulin) (Liu et al., 2016). However, it should be noted that Bivalent vaccine-SA-treatment exhibited no additive beneficial effect on T1DM prevention (in terms of morbidity and mortality) when compared with the monovalent BLPs vaccines-treatment (Figure 2). Thus, this interesting difference deserves further confirmation and investigation, which is vital for us to determine the best formulation of multivalent BLPs vaccines for future study.

As a simple test, the OGTT has been widely used in research and clinical practice to detect dysglycemia or help to diagnose diabetes. In addition, a recent study has shown that the OGTT owns the ability to predict T1DM, and thus it could be used for subject stratification in T1DM prevention trials (Baidal et al., 2022). In the OGTT performed in the current study, lower blood glucose levels at 15 min after glucose injection have been found in the bivalent BLPs vaccines-treated groups, compared with the monovalent BLPs vaccines-treated groups (Figure 3). Therefore, oral administration of the bivalent BLPs vaccines could endow the mice with a stronger blood glucose regulation ability. However, this blood glucose regulation ability was much weaker in the control groups (PBS group and BLPs group) as compared to the BLPs vaccines-treated groups (Figure 3). Thus, it may be suggested that inflammatory reactions around pancreatic islets have occurred in PBS and BLPs group at this time (21-week-old), and therefore a decline of the glucose sensitivity of β cells was induced (Siewko et al., 2014). The interplay of various pathogenic factors contributes to the initiation of these inflammatory reactions on pancreatic β cells during T1DM development, and β-cell dysfunction may exist years before T1DM diagnosis (Zajec et al., 2022; Evans-Molina et al., 2018). Therefore, theoretically, interventions such as immunomodulatory therapies should be applied as early as possible to maintain β-cell function and prolong β-cell insulin production (Mastrandrea & Quattrin, 2022). In the current study, oral administration of these BLPs vaccines significantly reduced the severity of insulitis compared with the control groups (Figure 4). Thus, treatment with these BLPs vaccines might suppress these islet inflammatory responses, and therefore preserving the integrity and function of pancreatic islets. Compared to the monovalent BLPs vaccines-treatment, Bivalent vaccine-mix-treatment exhibited a higher ability to preserve pancreatic islets and maintain β-cell insulin production (Figure 4), which may directly account for its superiority in preventing the onset of T1DM.

It has been suggested that antigen-induced oral tolerance might correlate with effector T cells clonal depletion and/or anergy, induction of Tregs, and immune deviation (Th1 to Th2) (Serra & Santamaria, 2019; Wambre & Jeong, 2018; Rezende & Weiner, 2022; Sricharunrat et al., 2018; Pinheiro-Rosa et al., 2021). Consistent with previous studies that antigen-specific Th2-related antibody subclasses could be induced when immunized orally with T1DM-associated autoantigens (Mao et al., 2019, 2022; Chen et al., 2018; Gong et al., 2010), oral administration of these BLPs vaccines induced the corresponding antigen-specific Th2-type (higher IgG1 levels) instead of Th1-type (higher IgG2a levels) humoral immune response (Figure 5). Importantly, compared to the monovalent BLPs vaccines-treatment, the bivalent BLPs vaccines-treatment could induce both SCI-59 and IA-2-specific IgG1 (Th2-associated isotype) (Figure 5). This could be further supported by the results obtained from the analysis of splenocyte proliferation and cytokine. Compared to splenocytes isolated from the monovalent BLPs vaccines-treated groups, the spontaneous proliferative reactivity to both SCI-59 and IA-2 was suppressed in splenocytes isolated from the bivalent BLPs vaccines-treated groups (Figure 6). In addition, treatment with the bivalent BLPs vaccines (especially Bivalent vaccine-mix) led to producing more Th2 cells but less Th1 cells than the monovalent BLPs vaccines treatment (Figure 7). This may have benefited from that the bivalent BLPs vaccines treatment induced a cytokine environment with more Th2 cytokines but less Th1 cytokines than those of the monovalent BLPs vaccines treatment (Figure 8). Because the local cytokine profile contributes to the differentiation of Th1/Th2 cells (Ruterbusch et al., 2020; Zhang et al., 2014), therefore, compared to the monovalent BLPs vaccines treatment, the cytokine environment containing more Th2 cytokines and less Th1 cytokines induced by additive effect of SCI-59 and IA-2 contained in the bivalent BLPs vaccines (especially Bivalent vaccine-mix) may more efficiently induce the differentiation of naïve T cells to Th2 cells, but not to Th1 cells. Taken together, compared to the monovalent BLPs vaccines treatment, the additive effect of SCI-59 and IA-2 was more beneficial for the correction of Th1/Th2 imbalance, which is one important factor responsible for the development of autoimmune disease, including T1DM (Crane & Forrester, 2005). Specific deletion of Foxp3^+^-induced Tregs completely abolished oral tolerance, and therefore, CD4^+^CD25^+^Foxp3^+^ Tregs contribute a lot to maintaining peripheral immune tolerance, and they are mandatory for induction of oral tolerance (Wing & Sakaguchi, 2010; Hadis et al., 2011). Therapeutic strategies which might expand Tregs in an antigen-dependent fashion may offer exciting avenues to induce tolerance in T1DM (Mitchell & Michels, 2022). Here, we observed that oral administration of the bivalent BLPs vaccines significantly increased the frequency of Tregs in PLNs compared to the monovalent BLPs vaccines treatment (Figure 9). Taken together, oral administration of these BLPs vaccines can prevent T1DM by correcting Th1/Th2 imbalance and inducing CD4^+^CD25^+^Foxp3^+^ Tregs. More importantly, an additive effect of both antigens contained in the Bivalent vaccine-mix on T1DM prevention was observed.

## Conclusion

In this study, various BLPs vaccines were constructed. Oral vaccination with these BLPs vaccines could suppress the onset of T1DM in NOD mice by stimulating antigen-specific tolerance. Importantly, compared to the monovalent BLPs vaccines, an additive beneficial effect on T1DM prevention was observed by oral treatment with the Bivalent vaccine-mix. Therefore, a combination of multiple autoantigens based on BLPs may be an effective approach to induce oral tolerance and prevent T1DM and other autoimmune diseases.
